# Methodological Assessment of ExoGAG for Isolation of Cerebrospinal Fluid Extracellular Vesicles as a Source of Biomarkers

**DOI:** 10.3390/ijms252413705

**Published:** 2024-12-22

**Authors:** Nil Salvat-Rovira, Anna Vazquez-Oliver, Elisa Rivas-Asensio, Marina Herrero-Lorenzo, Ana Gámez-Valero, Jesús Pérez-Pérez, Cristina Izquierdo, Antonia Campolongo, Eulàlia Martí, Jaime Kulisevsky, Rocío Pérez-González

**Affiliations:** 1 Movement Disorders Unit, Neurology Department, Hospital de la Santa Creu i Sant Pau, 08025 Barcelona, Spain; 2 Sant Pau Biomedical Research Institute (IIB-Sant Pau), 08041 Barcelona, Spain; 3 Centro de Investigación en Red-Enfermedades Neurodegenerativas (CIBERNED), 19171 Madrid, Spain; 4 Departament de Medicina, Universitat Autònoma de Barcelona, 08193 Bellaterra, Spain; 5 Department of Biomedicine, Faculty of Medicine, Institute of Neurosciences, University of Barcelona, 08036 Barcelona, Spain; 6 Instituto de Investigación Sanitaria y Biomédica de Alicante (ISABIAL), 03010 Alicante, Spain; 7 Instituto de Neurociencias, Universidad Miguel Hernández-CSIC, 03550 Sant Joan d’Alacant, Spain

**Keywords:** extracellular vesicles, cerebrospinal fluid, isolation method, ultracentrifugation, ExoGAG, biomarkers, miRNAs

## Abstract

Extracellular vesicles (EVs) in cerebrospinal fluid (CSF) represent a valuable source of biomarkers for central nervous system (CNS) diseases, offering new pathways for diagnosis and monitoring. However, existing methods for isolating EVs from CSF often prove to be labor-intensive and reliant on specialized equipment, hindering their clinical application. In this study, we present a novel, clinically compatible method for isolating EVs from CSF. We optimized the use of ExoGAG, a commercially available reagent that has been tested in plasma, urine and semen, and compared it directly with differential ultracentrifugation using Western blotting, protein quantification, nanoparticle tracking analysis, and cryogenic electron microscopy. Additionally, we analyzed the presence of specific microRNAs (miRNAs) known to be present in CSF-derived EVs. Our data demonstrate that ExoGAG is an effective method for isolating EVs from CSF, yielding a higher amount of EVs compared to traditional ultracentrifugation methods, and with comparable levels of specific miRNAs. In conclusion, the use of ExoGAG in a clinical setting may facilitate the testing of biomarkers essential for tracking brain pathology in CNS diseases.

## 1. Introduction

In diseases of the central nervous system (CNS), there is a pressing need to identify novel biomarkers that can aid with diagnosis, prognosis, and the assessment of brain-targeting interventions, such as those used in clinical trials. In the last decade, circulating extracellular vesicles (EVs) have emerged as a promising source of such biomarkers. EVs are membrane-enclosed vesicles composed of a phospholipid bilayer, secreted by cells into the extracellular space, and found in biological fluids. They contain proteins, lipids and nucleic acids that are specific to the cell of origin. Notably, EVs can transport proteins that aggregate during the course of neurodegenerative diseases, such as amyloid-β [1], tau [2], or huntingtin [3], and may constitute the vehicles for their spread [4]. Different species of EVs have distinct intracellular origins [5]. Exosomes, a type of EVs ranging from 20 to 150 nm in size, originate from the invagination of the late endosome/multivesicular body around cytoplasmic materials forming intraluminal vesicles that will be later exocytosed into the extracellular space. Alternatively, EVs can bud directly from the plasma membrane, giving rise to ectosomes. A third major type of EVs are apoptotic bodies, which are produced by cells undergoing apoptosis through plasma membrane blebbing or protrusion. Regardless of their origin, analyzing the composition of EVs can provide insights into the physiopathological state of the cells from which they originated, underscoring the potential of EVs as surrogate markers for brain-related processes [6]. Additionally, it has been established that all EV subtypes have distinct RNA profiles [7,8], including small non-coding RNAs (sRNAs) [9,10].

Because of its direct contact with the brain, cerebrospinal fluid (CSF), a clear and transparent fluid derived from blood plasma that lubricates the CNS [11], offers key insights into the state of CNS cells. Neurons, microglia, oligodendrocytes, and probably all brain cells release EVs into the interstitial space, from where they may reach the CSF [12,13]. Importantly, CSF is accessible via lumbar puncture, a procedure in which a needle is inserted between two lumbar vertebrae, facilitating the diagnosis of many neurological disorders. Indeed, several biomarkers in CSF have already been implemented in clinical practice, such as for Alzheimer’s disease diagnosis [14]. The amount of EVs present in CSF is very low compared to other biofluids, such as blood [15], making prior EV enrichment necessary to explore EVs as a source of biomarkers rather than analyzing the total CSF. In recent years, various isolation methods have been developed to efficiently separate EVs from the CSF, and, as in other biofluids, each method has trade-offs in terms of yield and purity of the EV preparations (reviewed in [16]). The use of sucrose or iodixanol density gradients with ultracentrifugation (UC) [17] and immunoisolation [18] methods lead to preparations with higher purity EVs but with a low EV yield, and a compromise between yield and purity can be obtained by differential UC [19,20] or Size-Exclusion Chromatography [21]. On the other hand, techniques such as precipitation [22] are known for producing high-yield but low-purity EVs, while methods like differential UC, which require ultracentrifuges, are often time-consuming, involve laborious procedures, and necessitate specialized equipment, making them unsuitable for clinical settings. In contrast, methods involving precipitation reagents require only a regular bench centrifuge and no specialized equipment. ExoGAG (Nexotech, Lugo, Spain) is a precipitation reagent that takes advantage of the presence of negatively charged glycosaminoglycans (GAGs) on the EV surface; the reagent binds to the GAGs and neutralizes their charge, forming a complex that can be purified by centrifugation [23,24]. Previous studies have demonstrated the efficiency of ExoGAG in isolating EVs from cell conditioned media [25], plasma [25], urine [26], and semen [27], but studies in CSF are lacking, unlike with other precipitating reagents [16]. In the present work, we describe a method to isolate EVs from CSF samples using ExoGAG and compare it head-to-head with UC. Additionally, given that microRNA (miRNA) species play critical roles in cellular regulation and may serve as biomarkers for CNS diseases [28], we evaluated the impact of the isolation method on the recovery of two recognized miRNAs present in CSF EVs. This investigation not only contributes to the optimization of EV isolation techniques but also holds the potential to enhance the utility of CSF EVs in biomarker discovery and clinical applications in CNS diseases.

## 2. Results

### 2.1. Evaluation of ExoGAG for CSF EV Isolation

**Figure 1:**
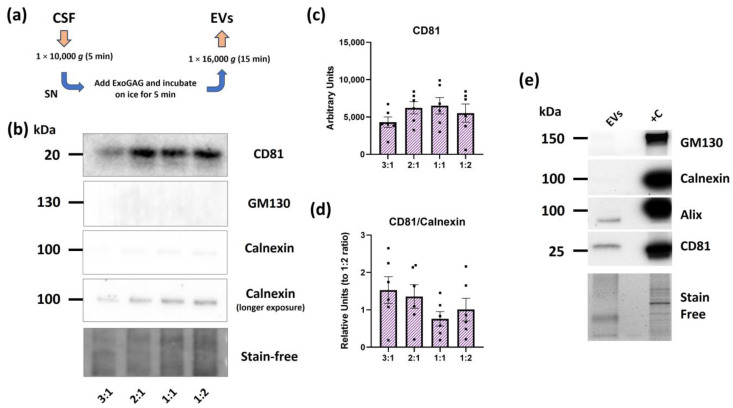
Effectiveness of ExoGAG in isolating EVs from CSF. (**a**) Schematic representation of the method used to precipitate EVs with ExoGAG. SN = supernatant. (**b**) Representative immunoblots of CD81 and the intracellular markers GM130 and calnexin, in EVs isolated with ExoGAG using different sample-to-reagent (CSF/ExoGAG) ratios. A longer exposure of calnexin is shown. Quantification of (**c**) CD81 and (**d**) CD81/calnexin ratio. CD81/calnexin levels were normalized to the average of the 1:2 ratio. *N* = 6 independent CSF samples. Data are represented as mean ± S.E.M. (**e**) Representative immunoblots of CD81 and Alix as EV markers, along with GM130 and calnexin in EVs isolated with ExoGAG when using the 2:1 sample-to-reagent ratio. Cell lysate was used as positive control (+C). Imaging of the stain-free membrane is shown for total protein in both Western blot panels.

In the initial phase of our study, we optimized a method to use ExoGAG in CSF (Figure 1a). We initially followed the provider’s instructions, which recommended using a sample-to-reagent ratio of 1:2 for other biofluids such as plasma or serum. By doing so, electron microscopy analysis revealed the presence of an excess of reagent that could interfere with the measurements. Therefore, we decided to decrease the amount of reagent while keeping the same starting volume of CSF (450 μL), to compare different CSF-to-reagent ratios (3:1, 2:1, 1:1, and 1:2). Although we did not detect any significant differences among ratios, we observed that a sample-to-reagent ratio of 2:1 and 1:1 yielded the highest amount of EVs, as indicated by the levels of CD81, a common EV-associated tetraspanin [29], by Western blot (Figure 1b,c). Western blot analysis also revealed that intracellular markers such as GM130 (Cis-Golgi) were mostly absent from all preparations (Figure 1b). For calnexin (endoplasmic reticulum), we observed very low immunoreactivity under standard exposure conditions (Figure 1b). To accurately assess calnexin levels and determine the ratio of ExoGAG to CSF that provides the best balance between vesicle yield and intracellular contamination, we extended the exposure time for better visualization. The results showed that the 2:1 ratio exhibited lower amounts of calnexin compared to the 1:1 and 1:2 ratios (Figure 1d), indicating reduced contamination by intracellular components. To further assess the purity of the 2:1 ratio, we explored the presence of Alix, alongside CD81, as EV markers, and confirmed the depletion of calnexin and GM130 when comparing with a cell lysate (Figure 1e). These results confirm that, when using a 2:1 ratio, ExoGAG precipitates EVs while minimizing contamination from intracellular components.

### 2.2. Head-to-Head Comparison of ExoGAG and Ultracentrifugation (UC) Methods

**Figure 2:**
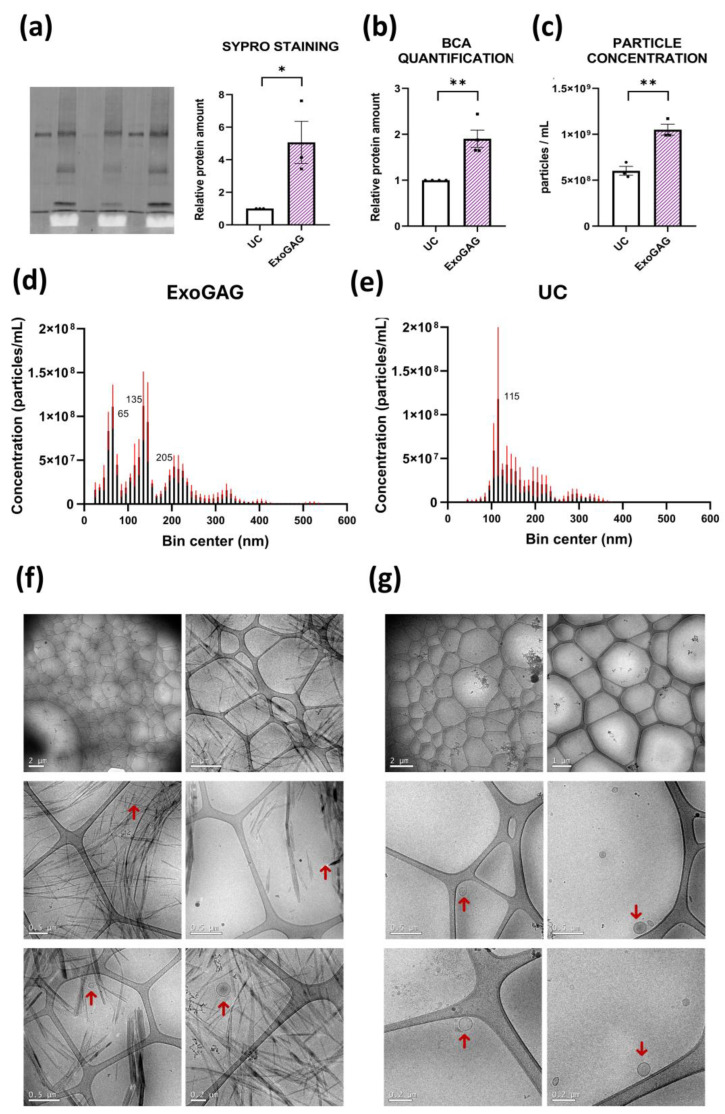
Comparative analysis of ExoGAG and UC isolated CSF EVs: protein estimation, NTA, and Cryo-EM. Quantification of total protein estimated by (**a**) Sypro staining or (**b**) BCA in EVs isolated with ExoGAG or UC. *N* = 3 for Sypro and *N* = 4 for BCA of independent CSF samples. Data are represented as mean ± S.E.M. (**c**) Comparison of the total particle (EVs) concentration after ExoGAG or UC isolation estimated by NTA. *N* = 3 independent CSF samples. Data are represented as mean ± S.E.M. * *p* < 0.05; ** *p* < 0.01 by Student’s *t*-test. Number and size profiling of EVs isolated with (**d**) ExoGAG or (**e**) UC by NTA. *N* = 3 independent EV samples per technique, each with three technical replicates (except for sample 1-UC, which only had two technical replicates). Data are represented as mean ± S.E.M. (shown as red lines). Cryo-EM images at different magnifications showing the presence of vesicles (indicated by red arrows) after using (**f**) ExoGAG at a 3:1 sample-to-reagent ratio or (**g**) UC. Calibration bars are indicated at the bottom of each image.

**Figure 3:**
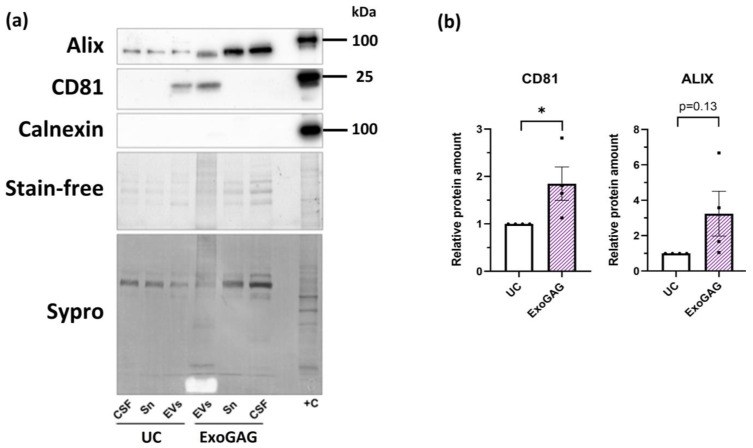
EV enrichment in ExoGAG-isolated versus UC-isolated EVs. (**a**) Representative immunoblots of EVs isolated using ExoGAG or UC, along with corresponding supernatants (Sn) and starting CSF. The absence of the intracellular marker calnexin in the EV preparations is also shown. Cell lysate (+C) was used as a positive control. The total amount of protein is shown in the stain-free gel and in the Sypro-stained membrane. (**b**) Quantification of CD81 and Alix levels in EVs isolated with ExoGAG or UC, based on *N* = 4 independent CSF samples. Data are represented as mean ± S.E.M. * *p* < 0.05 by Student’s *t*-test.

We conducted a direct comparative analysis of EV preparations isolated in parallel using ExoGAG and UC from the same starting CSF samples. First, we assessed the total amount of protein by two different techniques: Sypro staining (Figure 2a) and BCA (Figure 2b). Both techniques indicated a significantly higher total protein concentration in samples isolated with ExoGAG compared to those isolated with UC. These results are also reflected in the analysis of the number of particles detected in isolated EVs by Nanosight Tracking Analysis (NTA) (Figure 2c), where the particle count was higher with ExoGAG than with UC. The average concentration of EVs isolated with ExoGAG and UC was 1.05 × 10^9^ and 6.03 × 10^8^ particles/mL, respectively. Regarding the size of the particles, ExoGAG and UC showed different profiles. While with ExoGAG three different peaks were observed at 65, 135, and 205 nm (Figure 2d), with UC the majority of the particles had a size around 115 nm (Figure 2e). Cryogenic electron microscopy (Cryo-EM) analysis of ExoGAG preparations revealed the presence of abundant elongated structures, likely due to the precipitating reagent, which hindered the observation of EVs. Therefore, we moved to a sample-to-reagent ratio of 3:1 to better visualize the EVs for this specific analysis. The comparison between ExoGAG at a 3:1 ratio and UC showed that both methods yielded intact EVs with the expected shape and size (Figure 2f,g). Finally, Western blot analysis confirmed the presence of the EV markers CD81 and Alix in both conditions (Figure 3a). CD81 and Alix protein levels obtained with ExoGAG were normalized relative to those obtained with UC (Figure 3b). We loaded the same volume of EVs for each condition to maintain consistency and ensure a direct comparison of the EV yield between the two methods. Notably, CD81 levels were higher in ExoGAG-isolated EVs compared to those isolated by UC, with Alix levels also showing a trend towards an increase in ExoGAG-prepared EVs. EVs obtained using both methods showed depletion of intracellular components (Figure 3a). The observed enrichment of CD81 and Alix in the ExoGAG isolations can be attributed to the specific precipitation-based mechanism of this method, which may enhance the recovery of vesicles enriched in these markers compared to the UC protocol.

### 2.3. Evaluation of miRNA Cargo in EVs

**Figure 4:**
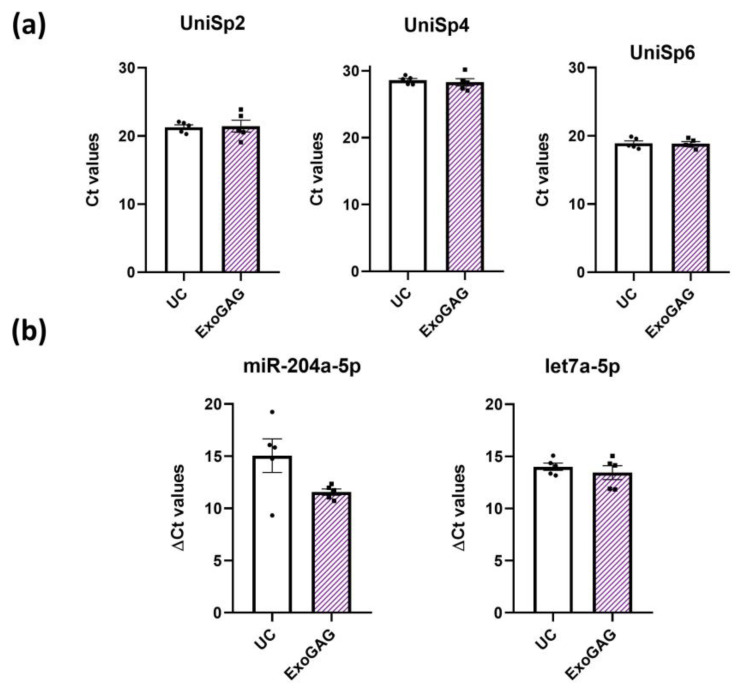
Presence of specific miRNAs in CSF EVs isolated by UC or ExoGAG. (**a**) Threshold cycle (Ct) values for the spike-in UniSp2, UniSp4, and UniSp6 in EVs isolated by UC and ExoGAG. (**b**) Delta Ct values for the determinations of miR-204a-5p and let7a-5p in EVs isolated by UC and ExoGAG. Data are represented as mean ± S.E.M., based on *N* = 5 independent CSF samples.

Next, to explore the cargo of EVs, we focused on identifying two miRNAs commonly found in EVs: miR-204a-5p and let-7a-5p [30]. We first verified that the isolation method did not interfere with downstream RNA extraction by including RNA spike-in controls in the EV preparations prior to extraction. We were particularly concerned that the ExoGAG reagent might interfere with the extraction columns. However, our data indicated that the efficiency of extraction was comparable in both methodologies, as indicated in the analysis of Ct in UniSp2 and UniSp4, as well as the RT reaction evaluated by the spike-in UniSp6 (Figure 4a). Furthermore, the quantification analysis of miR-204a-5p and let-7a-5p showed that the recovery of these two miRNAs with ExoGAG was similar (Figure 4b). These results indicate that ExoGAG does not adversely affect downstream RNA analysis.

## 3. Discussion

EVs in biofluids are gaining attention as potential sources of biomarkers for neurological disorders due to their ability to reflect brain pathology and cross the blood–brain barrier [31]. Their unique properties allow for non-invasive monitoring of CNS diseases, making them an attractive option for clinical applications. Specifically, the use of EVs derived from CSF in the context of neurological diseases offers distinct advantages over those derived from plasma or serum. While plasma and serum EVs are minimally invasive and easily accessible, they may not provide the same level of insight as CSF-derived EVs. Consequently, CSF-derived EVs hold significant potential as a source of biomarkers, particularly in diseases where lumbar puncture is already employed for diagnostic purposes. However, standardization of isolation methods and validation studies are essential for their successful translation into clinical practice. In the current work, we have tested the feasibility of using ExoGAG as a method to isolate EVs from CSF. After testing different sample-to-reagent ratios, we were able to pellet EVs enriched in canonical EV markers and mostly depleted of intracellular markers. In addition, we have compared ExoGAG efficacy with differential UC, a well-established method to separate EVs from biofluids and cell conditioned media [32]. In terms of protein and particle concentration, ExoGAG-prepared EVs exhibited higher numbers than UC EVs, indicating higher levels of EVs, which was supported by the higher levels of CD81 in ExoGAG preparations compared to UC. Regarding the size profile, ExoGAG yielded particles with an average size of 135 nm, similar to those precipitated by UC. However, we also detected smaller particles (65 nm) and, to a lesser extent, a population of larger particles (205 nm). The larger particles may indicate the presence of larger EVs or contamination with cell debris, which was not observed in the UC preparations. However, cell debris was not visible in the ExoGAG cryo-EM images. Instead, cryo-EM revealed the presence of filaments that co-precipitated with the EVs in ExoGAG samples, which might be problematic for some EV analysis or applications. To mitigate this, we tried to remove the filaments by passing the EV preparations through a 0.45 µm pore size filter. However, we observed significant loss of EVs after filtration, suggesting that they were likely bound to the reagent and unable to pass through the filter . Therefore, further research is necessary to determine whether the ExoGAG reagent can be effectively separated from the EVs for downstream applications.

In the ExoGAG conditions, we could detect a trend towards higher levels of Alix with ExoGAG. Alix (ALG-2 interacting protein X) is a canonical EV marker found in the luminal part of the vesicles [33], with a predicted molecular mass of 93 KDa. In our study, we mainly detected a 75 KDa band that was also present in CSF and supernatant. This band might correspond to a truncated form of Alix in which the C-terminal fragment is probably missing due to proteolysis [34]. It is worth noting that in the CSF EVs isolated by differential UC, we could also identify a faint band corresponding to Alix with the expected molecular size. Whether the truncated forms of Alix have biological significance in EVs needs to be further investigated.

Small RNAs such as miRNAs contained in EVs have shown high potential as biomarkers for neurological diseases, such as in Huntington’s [35] or Alzheimer’s [36] diseases. Our evaluation of the EV cargo, focusing on the miRNAs miR-204a-5p and let-7a-5p, confirmed that both ExoGAG and UC methods effectively isolated these RNAs without significant interference from the ExoGAG reagent. This reinforces ExoGAG as a reliable method for preserving RNA integrity, making it a viable option for future biomarker studies.

Other methodological alternatives for EV isolation in CSF include size exclusion chromatography (SEC) using custom-made or commercially available columns [37,38], and other precipitation reagents, such as those based on polyethyleneglicol [39]. In this study, we have demonstrated that ExoGAG is an additional viable alternative precipitation reagent for isolating EVs from CSF. The method has been standardized using an initial volume of 450 µL of CSF; however, we anticipate that this volume could be reduced, particularly when downstream EV analyses involve ultrasensitive techniques such as digital ELISA or RNA analysis. The optimization of isolation methods for low amounts of biological material is crucial, especially considering that existing biobanks typically provide limited volumes of CSF (from 0.2 to 0.5 mL). Given the advantages of analyzing CSF EVs over total CSF, ExoGAG could also be considered for biomarker studies during the discovery phase. Indeed, previous studies have shown the benefits of using CSF EVs versus total CSF in proteomic analyses, as the more abundant CSF proteins are removed during the isolation process [21,40]. Finally, we also have shown the advantages of ExoGAG over the UC method, which enables the analysis of higher yields of EVs from the same starting volume of CSF, thereby allowing for more comprehensive analyses per patient-derived sample. Although the UC method offers better purity compared to precipitation methods, we propose ExoGAG as a method of choice for high-throughput experiments. This approach would be particularly useful even when pilot data from UC are available, as it will support that the specific biomarkers are included within EVs.

## 4. Materials and Methods

### 4.1. Collection of CSF and EV Isolation

CSF was collected, centrifuged at 2000× *g* for 10 min, aliquoted in polypropylene tubes, and frozen at −80 °C until analysis. A starting volume of 450 µL of CSF was thawed on ice and EVs were isolated either by using the ExoGAG reagent (Nexotech, Lugo, Spain) or by UC. In the case of ExoGAG, we followed the manufacturer’s instructions for other biofluids with some modifications including the sample-to-reagent ratio and the conditions of the first centrifuge step to remove cell debris (10,000× *g* for 5 min instead of 2000× *g* for 10 min). For the optimization process, different sample-to-reagent ratios (3:1, 2:1, 1:1, and 1:2) were tested while keeping the rest of the parameters unchanged. The 2:1 ratio was used for the ExoGAG vs. UC comparison, except in the electron microscopy analysis, where a 3:1 ratio was applied. After centrifugation, supernatants were mixed with the reagent in a sample-to-reagent ratio of 2:1 (450 µL of CSF per 225 µL of ExoGAG), unless otherwise indicated, incubated on ice for 5 min and centrifuged at 16,000× *g* for 15 min at 4 °C. Then, the supernatant was carefully removed and the dark-blue pellet containing the EVs resuspended in the appropriate buffer. For UC, the samples were processed as described earlier [41] but using a smaller volume: briefly, CSF aliquots were subjected to subsequent centrifugation steps: 3500× *g* for 10 min, two times at 4500× *g* for 10 min, 10,000× *g* for 30 min and 100,000× *g* for 70 min. A small amount of EVs in PBS was used for nanoparticle tracking analysis and electron microscopy. The rest of the EVs were prepared for protein analysis as follows.

### 4.2. Sample Homogenization, Protein Estimation, and Western Blot Analyses

EVs in PBS were lysed in 2X RIPA buffer (1% *v*/*v* Triton X-100, 1% *w*/*v* sodium deoxycholate, 0.1% *w*/*v* SDS, 150 mM sodium chloride, 50 mM Tris-HCl pH 7.4, and 1 mM EDTA, all from Sigma-Aldrich, St. Louis, MO, USA) supplemented with protease inhibitors (Thermo Fisher Halt cocktail, Waltham, MA, USA), sonicated for 45 s in a cold bath, and kept on ice for 20 min with vigorous vortex every 3–4 min. The total protein levels of the EVs were estimated by using the Pierce BCA (bicinchoninic acid) Protein Assay Kit (Thermo Fisher Scientific, Waltham, MA, USA) according to the manufacturer’s protocol. Two μL of EVs in RIPA buffer (in duplicate) were incubated at 60 °C for 30 min in a polypropylene microplate (Corning Inc., Corning, NY, USA) with the BCA Reagent Solution (50:1 Reagent A to Reagent B, provided in the kit). A bovine serum albumin (BSA, provided in the kit) standard curve was loaded in parallel for each plate analyzed. The colorimetric reaction was quantified as the absorbance at 570 nm using the AD 340C microplate reader (Beckman Coulter, Indianapolis, IN, USA). The samples examined by Western blot analysis were supplemented with 4X Laemmli sample buffer (Bio-Rad, Hercules, CA, USA), boiled for 10 min at 90 °C, and equal volumes were loaded into 10% Tris-HCl gel electrophoresis (Stain-free criterion precast gels, Bio-Rad), and transferred onto PVDF membranes (Bio-Rad, Hercules, CA, USA) through a semi-dry system (Trans-blot turbo, Bio-Rad, Hercules, CA, USA). Sypro staining (Thermo Fisher Scientific, Waltham, MA, USA) to determine total protein content was performed in some membranes by activating with 7% acetic acid and 10% methanol solution for 15 min, then rinsing with water, and finally stained with Sypro for 15 min before imaging the membrane in a ChemiDoc XRS+ Imaging Systems (Bio-Rad, Hercules, CA, USA). Membranes were incubated with antibodies to Alix (1:1000, Cat# ABC40, Merck Millipore, Burlington, VT, USA), CD81 (1:1000, Cat# 56039, Cell Signaling Technologies, Danvers, MA, USA), GM130 (1:1000, Cat# 12480, Cell Signaling Technologies, Danvers, MA, USA) and calnexin (1:10,000, Cat# GTX109669, Genetex, Irvine, CA, USA). Total protein was imaged in the stain-free gels using the ChemiDoc XRS+, and the specific protein bands visualized through the GeneGnome XRQ chemiluminiscence imaging system after incubation of the blots for 5 min with Femto ECL and quantified using ImageJ software 1.53c version (NIH).

### 4.3. Nanoparticle Tracking Analyses (NTA)

The particle size distribution and concentration measurements were evaluated with a Nanosight NS300 (Particle Tracking Analysis) instrument (Malvern Panalytical, Malvern, UK). EVs were resuspended in 1X PBS and diluted to the working range of the system (10^6^–10^9^ particle/mL). Videos were captured and analyzed with the Nanosight NS300 software (version 3.4) using a sCMOS camera.

### 4.4. Cryogenic Electron Microscopy (Cryo-EM)

A total of 3.9 µL of EV suspension were blotted onto 400 mesh holey carbon grids (© Micro to Nano 2021) previously glow discharged in a PELCO easiGlow glow discharge unit. Next, the sample was plunged into liquid ethane (−180 °C) by means of a Leica EM GP cryo workstation and subsequently transferred and visualized in a Jeol JEM-2011 TEM electron microscope (Jeol Ltd., Tokyo, Japan) operating at 200 kV. Samples were maintained at −180 °C during their observation and captures were obtained with a Gatan CCD 895 ultrascan camera (Gatan Inc., Pleasanton, CA, USA). Image processing was performed using ImageJ software 1.53c version (NIH).

### 4.5. RNA Extraction

sRNA was isolated using the miRNeasy Serum/Plasma Advanced Kit (Qiagen, Venlo, The Netherlands) according to the manufacturer’s instructions. Prior to RNA isolation, all samples were adjusted to an input final volume of 200 µL through concentration using Amicon Ultra-2 100 KDa filters, and a synthetic RNA spike-in mix (UniSp2 and UniSp4, Qiagen) was added and used as quality control for the RNA isolation process, in accordance with the kit protocol instructions. RNA samples were eluted in 20 µL of RNAse-free water. Before the analysis, RNA was precipitated using glycogen (0.5 µg/µL, Roche, Basel, Switzerland), sodium acetate (3 M pH 4.8), and 100% ethanol. After 24 h at −20 °C, the mixtures were centrifuged at 14,000 rpm for 30 min at 4 °C. Carefully, the supernatant was removed, and the pellet was then washed with 75% ethanol followed by centrifugation at 12,000 rpm for 5 min at 4 °C. Finally, the supernatant was discarded, and the RNA pellet was dried at room temperature (RT) and dissolved at 65 °C for 3 min in 6 µL of RNAse-free water and stored maintaining the cold chain at −80 °C.

### 4.6. qPCR of miRNAs

Two μL of the isolated and purified RNA were employed for reverse transcription (RT) and cDNA synthesis using the miRCURY LNA RT Kit (Qiagen, Venlo, The Netherlands) according to the manufacturer’s instructions. Additionally, an RNA spike-in, UniSp6, was also included as a quality control for RT. The obtained cDNA was further diluted at 1:10 for qPCR, and 3 μL were used as a template in a 10 μL final volume reaction using the miRCURY LNA SYBR Green PCR Kit (Qiagen, Venlo, The Netherlands). We employed specific miRCURY LNA PCR assay primers (Qiagen, Venlo, The Netherlands) for miR-204a-5p and let7a-5p (Qiagen ID, YP00206072, sequence 5′-3′, TTCCCTTTGTCATCCTATGCCT; Qiagen ID, YP00205727, sequence 5′-3′, TGAGGTAGTAGGTTGTATAGTT). For each determination, reactions were performed in triplicate. qPCRs were carried out in a StepOnePlus Real-Time PCR System (Thermo Fisher Scientific, Waltham, MA, USA) using the cycling conditions specified by the manufacturer. The analysis of qRT-PCR data involves the analysis of cycles to threshold (Ct) values for the target miRNAs compared to the Ct values of the spike-in UniSp2 (delta Ct values) in each sample.

### 4.7. Statistical Analysis

Data were analyzed with GraphPad Prism Software (version 8.0) using unpaired Student’s *t*-test and Wilcoxon *t* testing. Comparisons were considered statistically significant when *p* < 0.05. Individual data were represented as single points in all figures.

## 5. Conclusions

The ExoGAG method presents a clinically applicable alternative to UC, offering higher EV yields and facilitating comprehensive analyses from the same volume of CSF. The advantages of ExoGAG in terms of simplicity, reduced equipment requirements, and efficiency make it a promising tool for biomarker discovery and analysis in diseases affecting the CNS. Future studies should explore the full range of biomarker applications using ExoGAG-isolated EVs and further validate its utility in clinical settings.

## Data Availability

The original contributions presented in the study are included in the article, further inquiries can be directed to the corresponding author.
